# Supplementary material for: Multiple Lines of Evidences Reveal Mechanisms Underpinning Mercury Resistance and Volatilization by Stenotrophomonas sp. MA5 Isolated from the Savannah River Site (SRS), USA
Source: Cells. 2019 Apr 3;8(4):309. doi: 10.3390/cells8040309 (PMC6523443; doi:10.3390/cells8040309)
Supplement: Supplementary file 1 [file cells-08-00309-s001.pdf]

Supplementary Materials

Multiple Lines of Evidences Reveal Mechanisms Underpinning Mercury Resistance and Volatilization by *Stenotrophomonas* sp. MA5 Isolated from the Savannah River Site (SRS), USA

Table S1: List of primers and probes used for qRT-PCR analysis

Target gene	Forward primer	Reverse primer	Probe
16s	CTCGGAATTACTGGGCGTAAAG	CGCTACCCTCTACCACATTCTA	AACGGACTTAAACGACCACCTACGC
merA	CCAAGTACGAAGGCATCCTG	TCGTTCAAACGGACGGTAAG	ACAACGGTGATGGCCGGATTACC
merD	GGTGAGCGTGCATATCGT	TCATCGAACAAGCCGTAGC	TACCTGCTGCGCGGATTGCT
merT	CAAACCGGGTGAGGTCTG	TGGCATGACGTAGGGAAATC	CCCAAGTGCGAGCTACTTACAAGCT
merE	CGTCCGAGACGCACAAA	CACGACAGCGAGGATGG	ACTGGCTGTGCTGACTTGCC
merR	CGTGCTGTCTGAGTTGGT	CTAAGGCATAGCCGAACCTG	AAGCTTGCTCCACCCTGTAGTGAC
merP	AGTTGACGTGACTTTCGAGAC	CTTGACGCTGGACGGATAG	ACCTTCGACGATGCCAAGACCAG

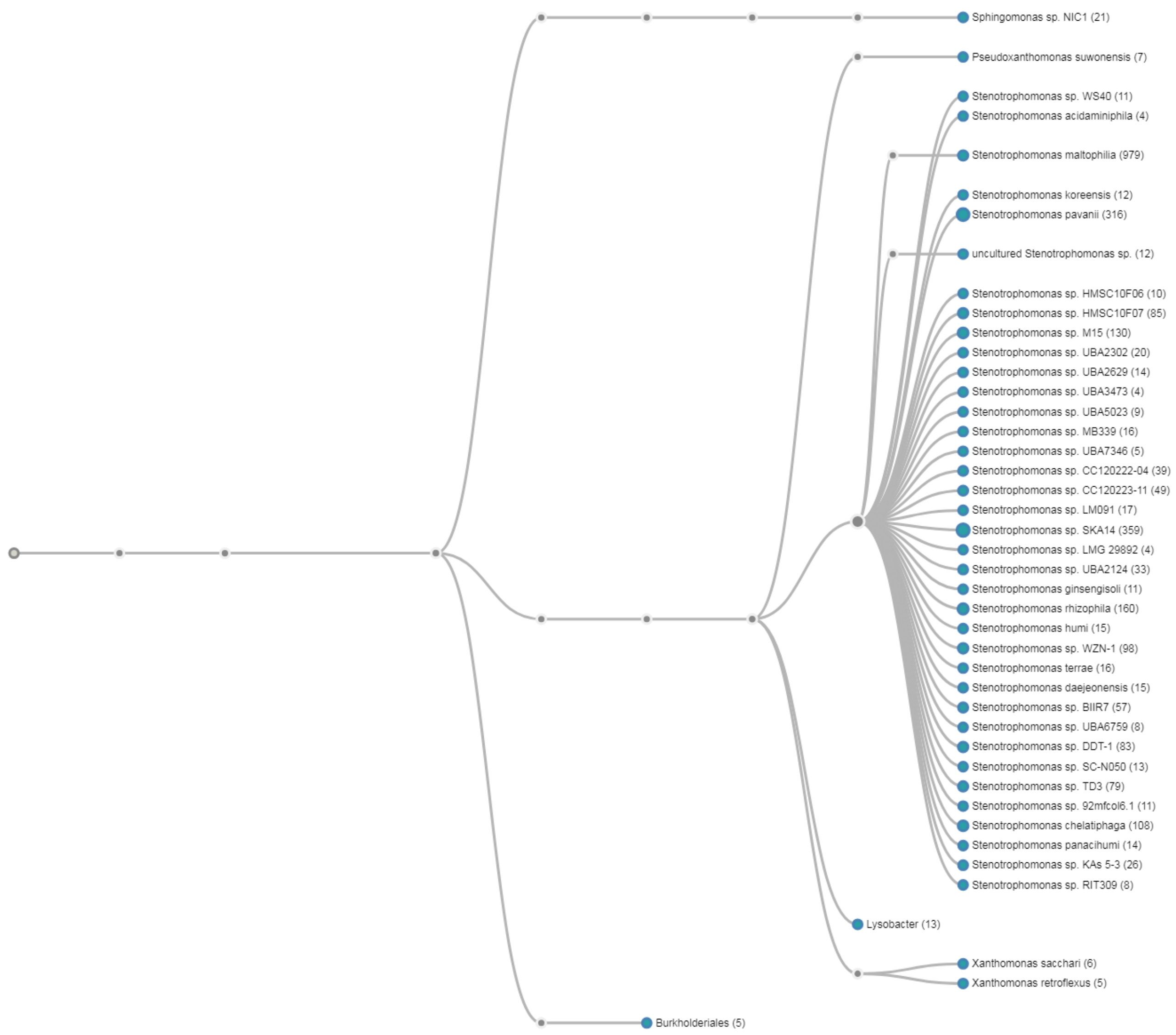

Figure S1: Whole genome sequence-based phylogenetic tress of isolated strain *Stenotrophomonas* sp. strain MA5. The highest taxonomic affiliation of strain was found with *Stenotrophomonas maltophilia* based on highest number of whole genome sequence reads (979; shown in parenthesis). Displayed nodes are those with at least 4 reads (<1% classified reads). This tree was generated by using one codex database.
